# Polysulfide Na_2_S_4_ regulates the activation of PTEN/Akt/CREB signaling and cytotoxicity mediated by 1,4-naphthoquinone through formation of sulfur adducts

**DOI:** 10.1038/s41598-017-04590-z

**Published:** 2017-07-06

**Authors:** Yumi Abiko, Yasuhiro Shinkai, Takamitsu Unoki, Reiko Hirose, Takashi Uehara, Yoshito Kumagai

**Affiliations:** 1 Faculty of Medicine, University of Tsukuba, Tsukuba, Ibaraki 305-8575 Japan; 2 Department of Medicinal Pharmacology, Graduate School of Medicine, Dentistry, and Pharmaceutical Sciences, Okayama University, Okayama, 700-8530 Japan

## Abstract

Electrophiles can activate redox signal transduction pathways, through actions of effector molecules (*e.g*., kinases and transcription factors) and sensor proteins with low p*K*a thiols that are covalently modified. In this study, we investigated whether 1,4-naphthoquinone (1,4-NQ) could affect the phosphatase and tensin homolog (PTEN)–Akt signaling pathway and persulfides/polysulfides could modulate this adaptive response. Simultaneous exposure of primary mouse hepatocytes to Na_2_S_4_ and 1,4-NQ markedly decreased 1,4-NQ-mediated cell death and *S*-arylation of cellular proteins. Modification of cellular PTEN during exposure to 1,4-NQ was also blocked in the presence of Na_2_S_4_. 1,4-NQ, at up to 10 µM, increased phosphorylation of Akt and cAMP response element binding protein (CREB). However, at higher concentrations, 1,4-NQ inhibited phosphorylation of both proteins. These bell-shaped dose curves for Akt and CREB activation were right-shifted in cells treated with both 1,4-NQ and Na_2_S_4_. Incubation of 1,4-NQ with Na_2_S_4_ resulted in formation of 1,4-NQ–S–1,4-NQ-OH. Unlike 1,4-NQ, authentic 1,4-NQ-S-1,4-NQ-OH adduct had no cytotoxicity, covalent binding capability nor ability to activate PTEN-Akt signaling in cells. Our results suggested that polysulfides, such as Na_2_S_4_, can increase the threshold of 1,4-NQ for activating PTEN–Akt signaling and cytotoxicity by capturing this electrophile to form its sulfur adducts.

## Introduction

There are a variety of cellular redox signaling processes, involving sensor proteins with low p*K*a thiol groups and effector molecules (*e.g*., kinases and transcription factors). Chemical modification of the sensor proteins through their thiol groups is believed to be associated with substantial activation of the effector molecules^1–6^. Examples of sensor molecules that can undergo *S*-modification include kelch-like ECH-associated protein 1 (Keap1), protein tyrosine phosphatase 1B (PTP1B), heat shock protein 90 (HSP90) and phosphatase and tensin homolog (PTEN). Their respective effector molecules, nuclear factor (erythroid-derived 2)-like 2 (Nrf2), epidermal growth factor receptor (EGFR), heat shock factor 1 (HSF1) and Akt, are, thereby, activated, leading to upregulation of their downstream genes^7–9^. Endogenous electrophiles generated during oxidative stress and inflammation, such as nitrated fatty acids and 8-nitro-cyclic guanosine monophosphate, can modify sensor proteins as well, resulting in activation of these redox signaling pathways^10, 11^.

Accumulated evidence has indicated that environmental electrophiles can also modulate redox signaling. For example, 1,4-naphthoquinone (1,4-NQ), 1,2-NQ and *tert*-butyl-1,4-benzoquinone activated the Keap1–Nrf2 pathway and 1,2-NQ activated the PTP1B–EGFR signaling pathway^9, 12–15^. Electrophilic organometallic methylmercury (MeHg) activated the PTEN–Akt–CREB signaling pathway at lower concentrations, through *S*-mercuration of PTEN. At higher concentrations, MeHg disrupted this redox signaling though nonspecific *S*-mercuration of CREB^16^. However, activation of PTEN–Akt–CREB signaling by quinones is still not well understood. Collaborating with Nishida *et al*., we reported that endogenous and exogenous electrophiles activated the H-Ras signaling pathway, through *S*-modification of H-Ras, and that this electrophilic signaling was negatively regulated by hydrogen sulfide (H_2_S) produced by cystathionine γ-lyase (CSE), and cystathionine β-synthase (CBS)^17^. These observations suggested that not only H-Ras signaling, but also other redox signaling pathways that can be activated by electrophiles, are potentially modulated by sulfur species.

We recently found that overexpression and knockdown of CBS and CSE enhanced and decreased, respectively, activation of the HSP90–HSF1 signaling pathway by environmental electrophiles such as cadmium (Cd) and 1,4-NQ^18, 19^. In addition, 1,4-NQ-induced activation of the HSP90–HSF1 signaling pathway in A431 cells was significantly suppressed by simultaneous treatment with the persulfide and polysulfide models, sodium disulfide (Na_2_S_2_) and sodium tetrasulfide (Na_2_S_4_)^19^. Moreover, in a cell-free study, the enzymatic reaction of CSE in the presence of 1,4-NQ produced 1,4-NQ–sulfur adducts such as 1,4-NQ–S–1,4-NQ [(1,4-NQ)_2_S], also produced by reaction of 1,4-NQ with H_2_S^19^. From these observations we hypothesized that reactive per/polysulfides, could suppress 1,4-NQ-mediated modulation of HSP90–HSF1 signaling, by capturing 1,4-NQ involved in its sulfur adduct formation. In this study, we investigated activation of the PTEN–Akt signaling pathway by 1,4-NQ and the contributions of persulfides/polysulfides to modulation of this signaling.

## Results

### Suppression of cytotoxicity and covalent modification of cellular proteins mediated by 1,4-NQ, with and without Na_2_S_4_

**Figure 1:**
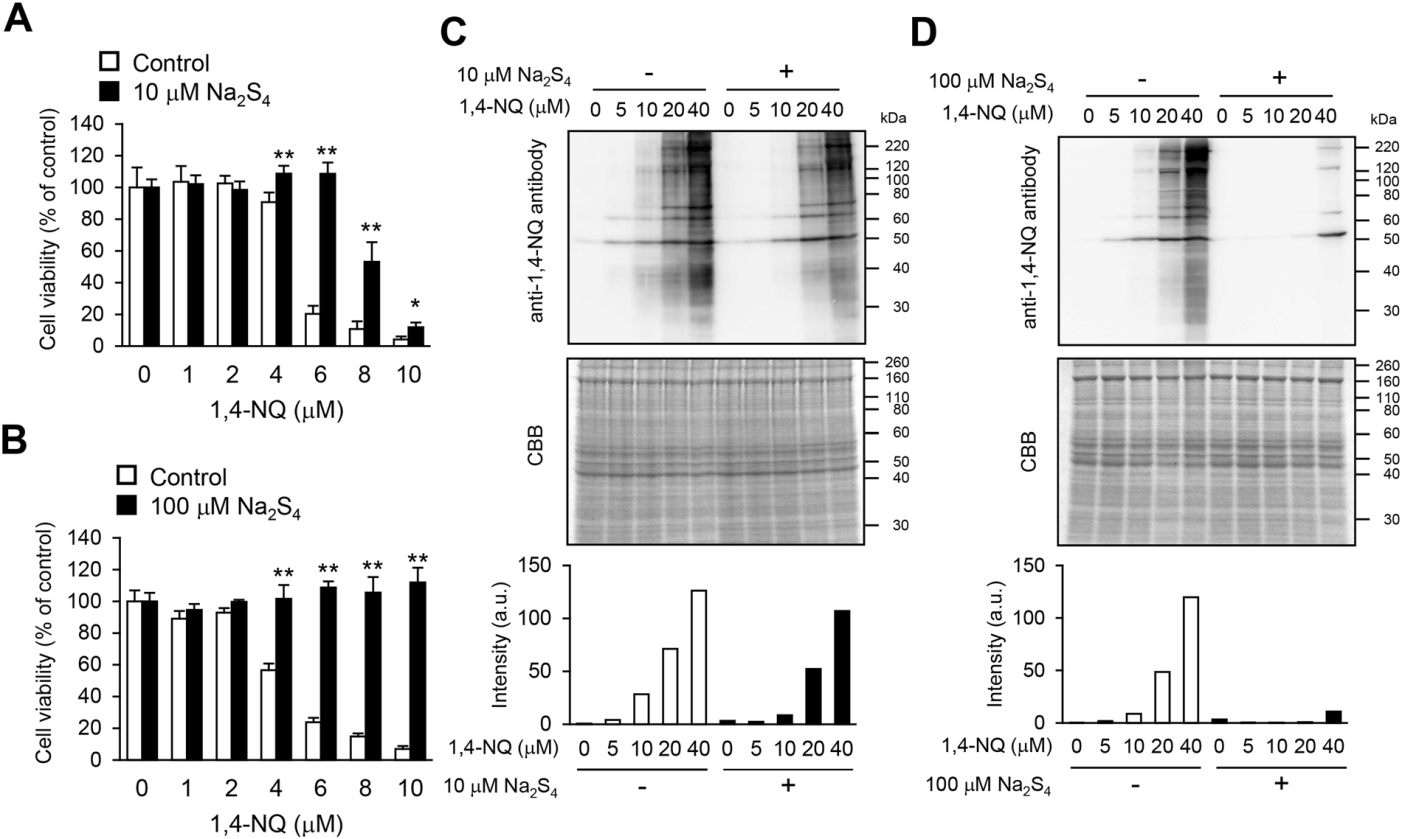
Cytotoxicity and chemical modification of cellular proteins during treatment of cells with 1,4-NQ, with or without Na_2_S_4_. (**A**,**B**) Primary mouse hepatocytes were exposed to 1,4-NQ with or without 10 (**A**) or 100 (**B**) µM Na_2_S_4_ for 24 h. Cell viability was assessed with the MTT assay. Each value is the mean ± standard error for three independent experiments. *P < 0.05 and **P < 0.01, compared with the control. C, D: Primary mouse hepatocytes were exposed to 1,4-NQ, with or without 10 (**C**) or 100 (**D**) µM Na_2_S_4_ for 1 h. Covalent modification of cellular proteins by 1,4-NQ was detected by western blotting using an anti-1,4-NQ antibody (upper), proteins were detected following SDS-PAGE by Coomassie Brilliant Blue staining (middle) and intensities of modified protein bands were calculated with Multi Gauge software (lower). Representative data are shown from two independent experiments.

Exposure of primary mouse hepatocytes to 1,4-NQ caused concentration dependent cell death and covalent modification of cellular proteins, the latter indicated by western blotting with a specific antibody against 1,4-NQ (Fig. 1)^20^. Simultaneous exposure to 1,4-NQ (10 µM) with Na_2_S_4_ (10 µM) significantly decreased 1,4-NQ-dependent cytotoxicity (Fig. 1A), whereas, at 100 µM, Na_2_S_4_ completely prevented the toxicity (Fig. 1B). Cytotoxicity mediated by electrophiles is reportedly caused, at least in part, by nonspecific modification of cellular proteins^13^. Simultaneous treatment with Na_2_S_4_, however, suppressed protein modification by 1,4-NQ (Fig. 1C and D).

### Modulation of 1,4-NQ-mediated activation of PTEN–Akt signaling by Na_2_S_4_

**Figure 2:**
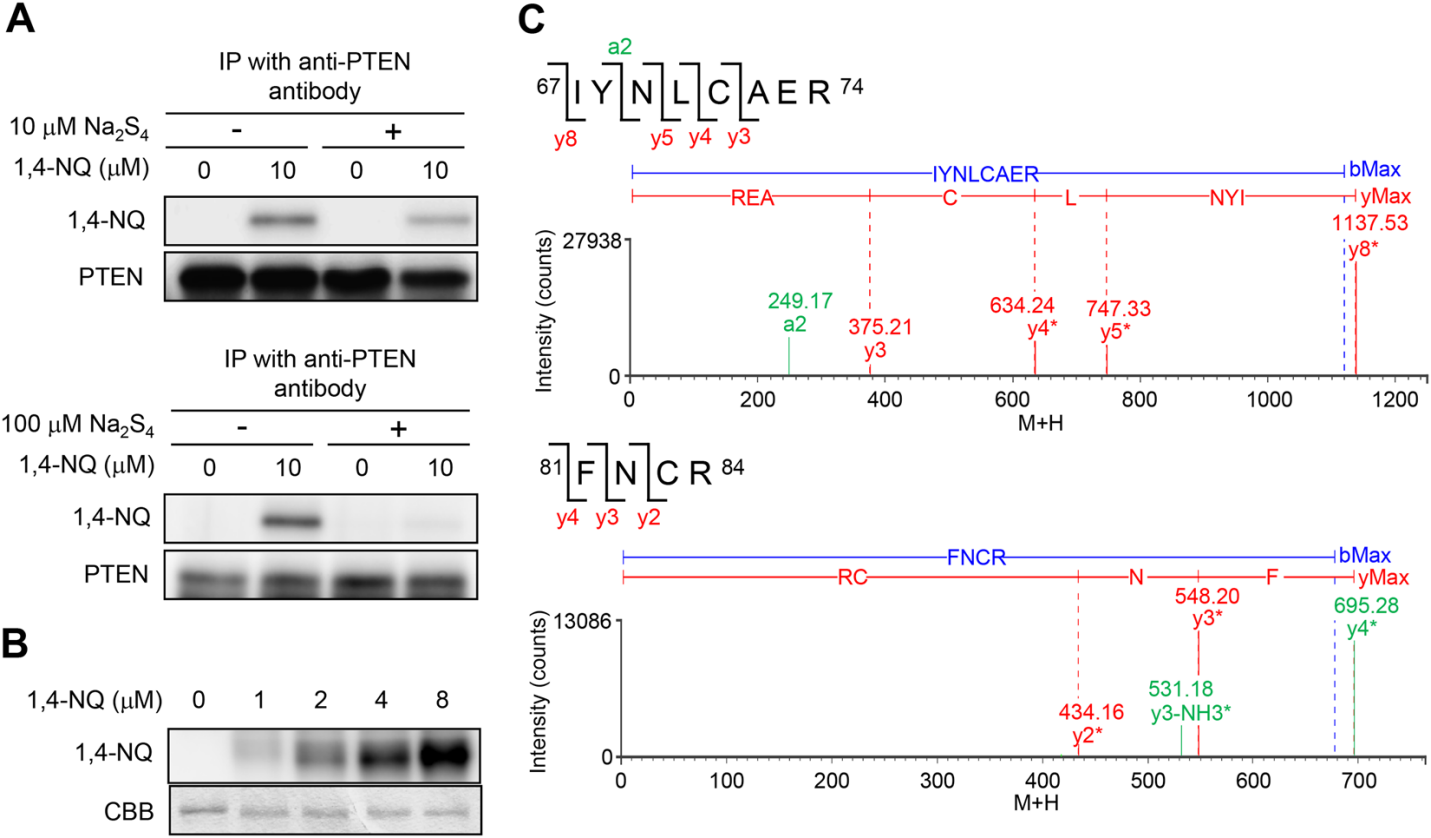
Chemical modification of cellular and recombinant PTEN by 1,4-NQ and suppression of 1,4-NQ modification of cellular PTEN. (**A**) Primary mouse hepatocytes were simultaneously treated with DMSO or 1,4-NQ and 10 (upper) or 100 (lower) µM Na_2_S_4_ for 30 min, then PTEN was immunoprecipitated using anti-PTEN antibody. Western blotting was performed using the indicated antibodies. Representative blots are shown from three independent experiments. (**B**) Recombinant GST-tagged human PTEN (1 μg) was incubated with 1,4-NQ (1–8 μM) at 25 °C for 1 h. The reaction mixture was then subjected to immunoblotting, with the anti-1,4-NQ antibody, and SDS-PAGE, with Coomassie Brilliant Blue staining. Representative blots are shown from three independent experiments. (**C**) Results of nanoUPLC-MS^E^ analysis of 1,4-NQ-modified cysteine residues in GST-tagged human PTEN. Recombinant GST-tagged human PTEN (1.7 μg) was incubated with 1,4-NQ (10 μM) at 25 °C for 30 min in a total volume of 10 μL 50 mM Tris-HCl (pH 7.5). After the reaction, PTEN protein was digested with trypsin and analyzed by nanoUPLC-MS^E^. The corresponding MS^E^ data are shown in Table 1.

*S*-Arylation of PTEN by 1,4-NQ in primary mouse hepatocytes was detected after immunoprecipitation of the PTEN. As shown in Fig. 2A, PTEN modification was observed after exposure of cells to 10 µM 1,4-NQ for 30 min. Under conditions corresponding to Fig. 1C and D, Na_2_S_4_ inhibited the covalent modification of PTEN by 1,4-NQ (Fig. 2A). Recombinant human PTEN (0.73 µM) was also modified by 1,4-NQ in a concentration dependent manner (Fig. 2B). It is well recognized that 1,4-NQ undergoes *S*-arylation to proteins through an 1,4-addition reaction by nucleophiles, resulting in formation of 1,4-NQH_2_ (MW = 158.02)-protein adduct^13^. We recently observed that this adduct readily underwent autooxidation, to yield a 1,4-NQ (MW = 156.02)-protein adduct^19^. Consistent with this, the trypsinized fragments detected by ultra-performance liquid chromatography (UPLC)-mass spectrometry (MS) in recombinant PTEN that had been modified by 1,4-NQ were 1,4-NQ-protein, not 1,4-NQH_2_-protein, adducts. We also found that the PTEN sites modified by 1,4-NQ were Cys71 and Cys83 (Fig. 2C and Table 1). This suggested that 1,4-NQ activated Akt signaling through *S*-arylation to PTEN and that Na_2_S_4_ suppressed this 1,4-NQ-mediated activation of the PTEN–Akt signaling pathway.

**Table 1:** MS^E^ data for 1,4-NQ-modified peptides in human PTEN.

position	assignment	calculated mass (Da)	observed mass (Da)	analyte modifier
61–74	a2	249.16	249.17	+1,4-NQ C (1)
	y3	375.20	375.21	
	y4*	634.23	634.24	
	y5*	747.31	747.33	+1,4-NQ C (1)
	y6*	1137.5	1137.53	+1,4-NQ C (1)
81–84	y2*	434.15	434.16	+1,4-NQ C (1)
	y3*	548.20	548.20	+1,4-NQ C (1)
	y2-NH_3_*	417.12	417.12	+1,4-NQ C (1)
	y3-NH_3_*	531.17	531.18	+1,4-NQ C (1)
	y4 *	695.26	695.28	+1,4-NQ C (1)

Recombinant GST-tagged human PTEN (1.7 μg) was incubated with 1,4-NQ (10 μM) at 25 °C for 30 min in 50 mM Tris-HCl (pH 7.5). After the reaction, the GST-PTEN protein was digested by trypsin and analyzed by nanoUPLC-MS^E^. The mass number 156.02 was used to calculate 1,4-NQ modification.

**Figure 3:**
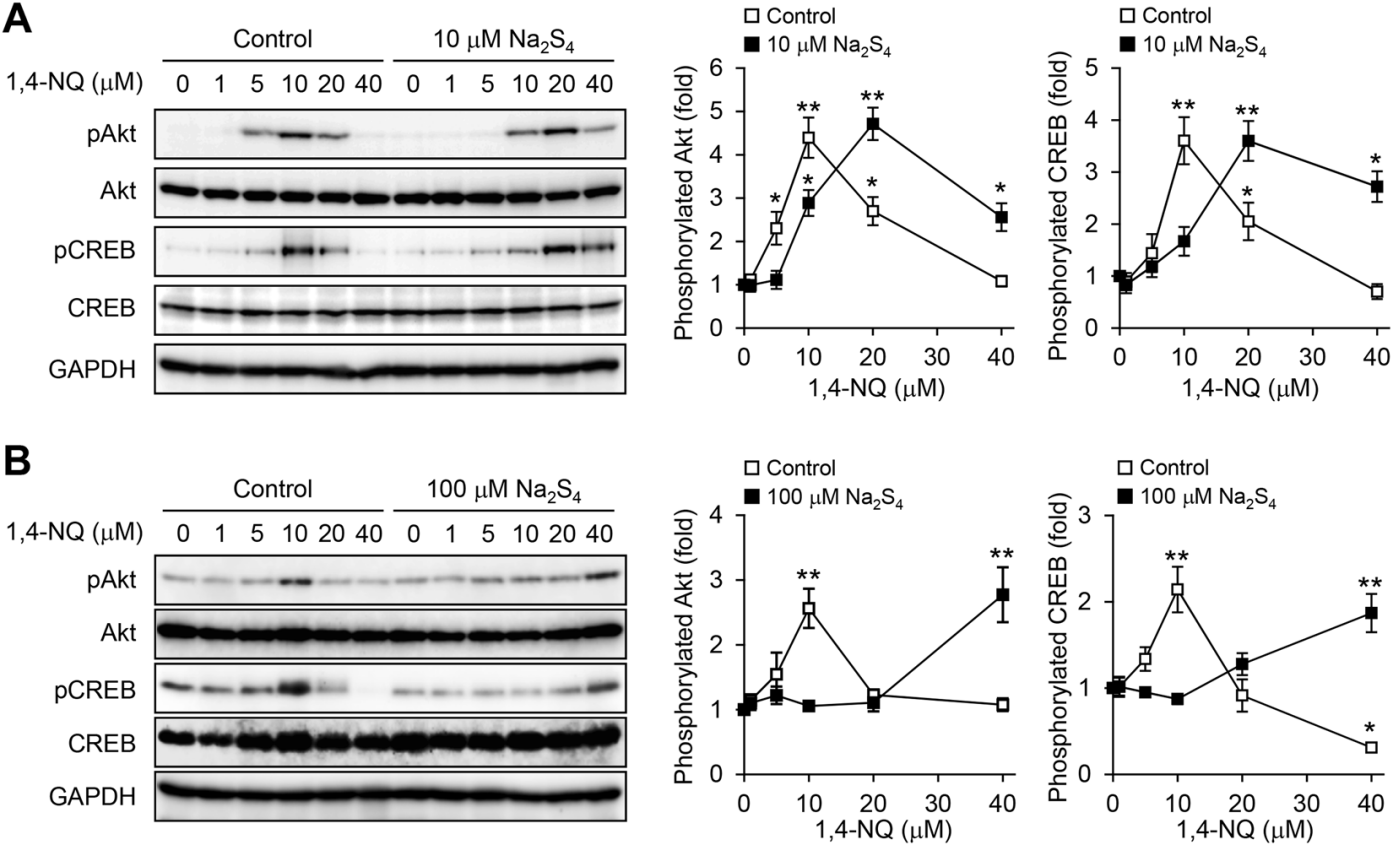
Participation of polysulfide in the 1,4-NQ-mediated phosphorylation of Akt and CREB in primary mouse hepatocytes. (**A**) Cells were exposed to the indicated concentrations of 1,4-NQ for 30 min in the presence of Na_2_S_4_ at 0, 10 (**A**), or 100 (**B**) µM. Akt and CREB phosphorylation was determined by western blotting (right). Representative blots are shown from three independent experiments. Band intensities were normalized to those of total Akt and CREB, respectively (left). Intensities are presented as fold induced relative to results with 0 µM 1,4-NQ exposure. Each value is the mean ± standard error for three independent experiments. *P < 0.05 and **P < 0.01, compared with 0 µM 1,4-NQ exposure.

To address whether 1,4-NQ would activate Akt signaling and Na_2_S_4_ would modulate this effect, we exposed primary mouse hepatocytes to 1,4-NQ, with or without Na_2_S_4_, and then measured phosphorylation of Akt and its downstream protein CREB by western blotting. As shown in Fig. 3, 1,4-NQ, at up to 10 µM, increased phosphorylation of Akt and CREB in a concentration dependent manner. However, phosphorylation, hence activation, of both proteins was inhibited by 1,4-NQ at higher concentrations. The bell-shaped 1,4-NQ dose responses for effects on this redox signal transduction pathway agreed with our findings that MeHg, at lower concentrations, activated PTEN–Akt–CREB signaling through *S*-modification of PTEN and, at higher concentrations, disrupted the cascade through non-specific *S*-modification of CREB^16^. At 10 µM, Na_2_S_4_ significantly decreased 1,4-NQ induced phosphorylation of Akt and CREB (Fig. 3A). Treatment with 1,4-NQ and 100 µM Na_2_S_4_ together led to a markedly right-shifted bell-shaped response curves for Akt and CREB phosphorylation (Fig. 3B), relative to those obtained with 1,4-NQ alone.

### Determination of 1,4-NQ-sulfur adducts formed by reaction of 1,4-NQ with Na_2_S_4_

**Figure 4:**
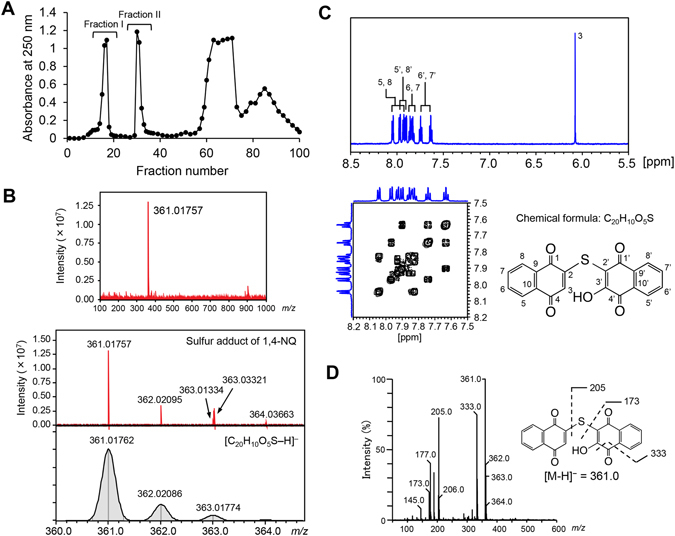
Purification and identification of the sulfur adduct of 1,4-NQ. (**A**) Separation of sulfur adducts of 1,4-NQ by column chromatography. 1,4-NQ (5 mM) was incubated with Na_2_S_4_ (10 mM) for 10 min at room temperature. The resulting solution was applied to an ODS column and eluted with 20% acetonitrile for 40 min followed by 80% acetonitrile for 60 min at a flow rate of 10 mL/min. Each fraction was analyzed by UV absorbance at 250 nm and by UPLC-MS. Fraction II primarily contained *m/z* 361 in negative ion mode. (**B**) FT-ICR-MS of the purified sulfur adduct. ESI-MS spectrum of the reaction product with *m/z* 361 (upper) and comparison of isotope ratios between the product and an elemental composition of C_20_H_10_O_5_S (lower). (**C**) Magnified views of the ^1^H NMR (upper) and ^1^H-^1^H COSY NMR (lower) spectra of a sulfur adduct of 1,4-NQ with *m/z* 361. Four doublet proton signals at δ 8.05 (d, J = 3.7 Hz, 1 H), 7.97 (d, J = 3.6 Hz, 1 H), 7.93 (d, J = 3.7 Hz, 1 H) and 7.91 (d, J = 5.8 Hz, 1 H), and four triplet proton signals at δ 7.86 (t, J = 7.4 Hz, 1 H), 7.83 (t, J = 7.4 Hz, 1 H), 7.74 (t, J = 7.5 Hz, 1 H) and 7.63 (t, J = 7.5 Hz, 1 H) were detected. An additional singlet proton signal in the high field at δ 6.07 (s, 1 H) must be attributable to H-3. An aromatic OH group must be located at the C-3′ position, although this OH signal was not detected. The COSY NMR spectrum showed that two triplets at δ 7.74 and δ 7.63 ppm were correlated to one another and to two doublets at δ 7.97 and δ 7.9 ppm, respectively. These signals must be attributable to H-5′, H-6′, H-7′ and H-8′. The other two triplets at δ 7.86 and δ 7.83 ppm were correlated to one another and to two doublets at δ7.93 and δ 8.05 ppm, respectively. These signals must be attributable to H-5, H-6, H-7 and H-8. D: MS spectrum of the sulfur adduct (*m/z* 361) of 1,4-NQ formed during incubation with Na_2_S_4_. The purified sulfur adduct was analyzed by UPLC-MS. Representative data are shown from three independent experiments.

We recently found that incubation of 1,4-NQ with Na_2_S_4_ consumed 1,4-NQ, suggesting that 1,4-NQ can react with Na_2_S_4_ to form sulfur adducts. Such reaction products might then become less reactive with primary mouse hepatocytes because of their decreased electrophilicity. To test this hypothesis, the sulfur adducts formed in a reaction mixture of 1,4-NQ with Na_2_S_4_ were separated on a preparative ODS-column, eluted with 20% acetonitrile and monitored spectrophotometrically at 250 nm. Fractions ranging from 14 to 18 min (Fraction I) and from 30 to 33 min (Fraction II) were collected (Fig. 4A) and reaction products were analyzed in each. The molecular masses of the reaction products in Fractions I and II were mainly *m/z* 345 and 361, respectively, by UPLC-MS analysis. This suggested that they were identical to 1,4-NQ–S–1,4-NQ (C_20_H_10_O_4_S) and 1,4-NQ–S–1,4-NQ-OH (C_20_H_10_O_5_S) adducts. The purified product with *m/z* 361 was analyzed by FT-ICR-MS to confirm its elemental composition as C_20_H_10_O_5_S (Fig. 4B), because we could not produce enough highly purified reaction product from Fraction I to identify it. As shown in Figs 4C, S1,S2 and S3, NMR analysis indicated a product that was structurally identical to 2-[(1,4-dioxonaphthalen-2-yl)sulfanyl]-3-hydroxynaphthalene-1,4-dione (1,4-NQ–S–1,4-NQ-OH). The MS fragmentation pattern of the product also supported this structure (Fig. 4D).

### Characterization of the 1,4-NQ–S–1,4-NQ-OH adduct

**Figure 5:**
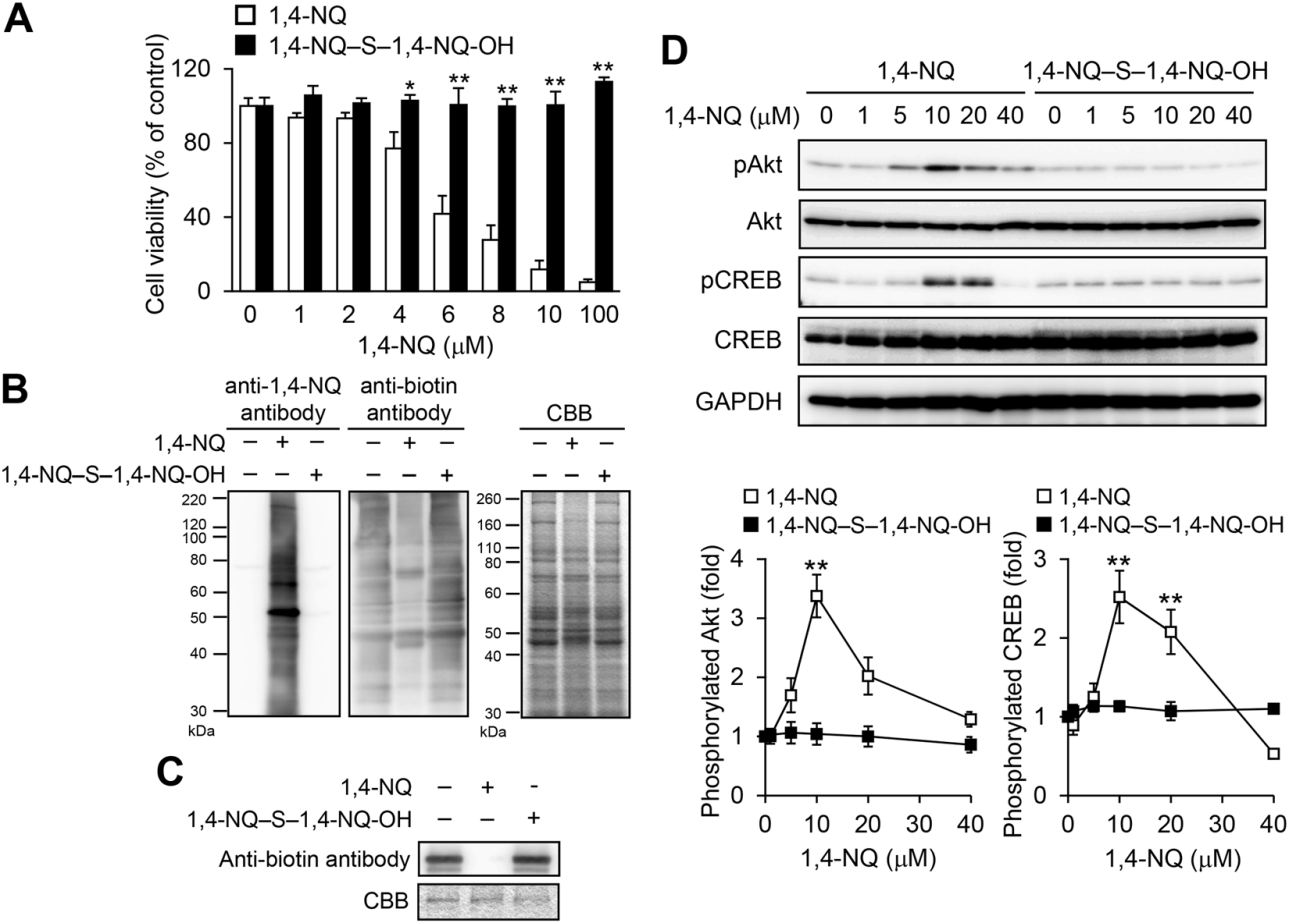
Cytotoxicity, covalent protein modification and Akt–CREB signaling activation caused by exposure of primary mouse hepatocytes to 1,4-NQ-sulfur adducts. (**A**) Primary mouse hepatocytes were exposed to 1,4-NQ or 1,4-NQ–S–1,4-NQ-OH for 24 h. Cell viability was determined with the MTT assay. Each value is the mean ± standard error for three independent experiments. (**B**) Cells were exposed to 1,4-NQ–S–1,4-NQ-OH (20 µM) or 1,4-NQ (40 µM) for 1 h. Covalent modification of cellular proteins with 1,4-NQ, as detected by western blotting (left), BPM assay results (middle) and protein bands on SDS-PAGE, as stained by Coomassie Brilliant Blue (right). Representative blots are shown from three independent experiments. (**C**) Recombinant GST-tagged human PTEN (1 μg) was incubated with 1,4-NQ–S–1,4-NQ-OH (5 µM) or 1,4-NQ (10 µM) at 25 °C for 1 h and then further incubated with BPM (15 μM) at 37 °C for 30 min. The reaction mixture was then analyzed by immunoblotting with the anti-biotin antibody and Coomassie Brilliant Blue was used to visualize bands after SDS-PAGE. Representative blots are shown from three independent experiments. (**D**) Cells were exposed to different concentrations of 1,4-NQ-sulfide for 30 min. Akt and CREB phosphorylation was determined by western blotting (upper). Representative blots are shown from three independent experiments. Band intensities were normalized to those for total Akt and CREB, respectively (lower). Intensities are expressed as fold induced, relative to results with 0 µM 1,4-NQ exposure. Each value is the mean ± the standard error for three independent experiments. *P < 0.05 and *P < 0.01, compared with the 0 µM 1,4-NQ exposure.

Exposure of primary mouse hepatocytes to authentic 1,4-NQ–S–1,4-NQ-OH adduct led to neither cytotoxicity nor *S*-arylation of cellular proteins, at up to 100 and 20 µM, respectively (Fig. 5A and B). PTEN was also not modified by the 1,4-NQ sulfur adduct (Fig. 5C). Under these conditions, phosphorylation of Akt or CREB was not detected in cells exposed to the 1,4-NQ-sulfur adduct, unlike in those exposed to 1,4-NQ (Fig. 5D).

## Discussion

**Figure 6:**
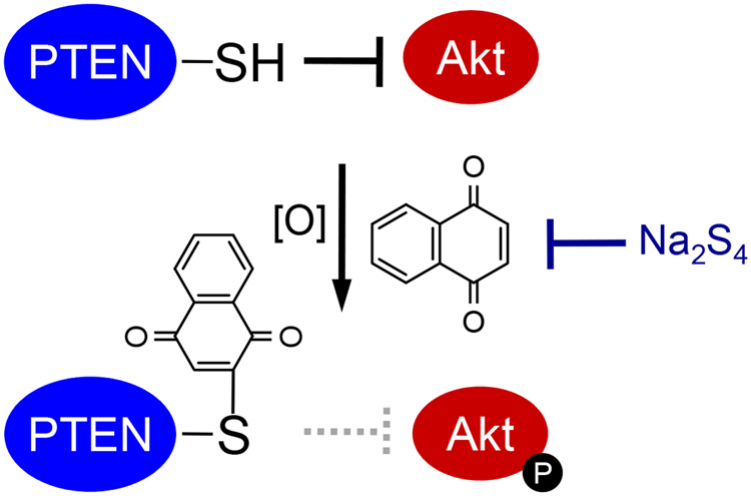
1,4-NQ-mediated activation of PTEN–Akt signaling, which was suppressed by Na_2_S_4_.

In our study, the atmospheric electrophile 1,4-NQ activated PTEN–Akt signaling at lower concentrations but disrupted it at higher concentrations. In addition, 1,4-NQ-mediated redox signaling was negatively regulated by a model polysulfide, Na_2_S_4_, through formation of 1,4-NQ sulfur adducts (Fig. 6). Under basal conditions, PTEN can negatively regulate the Akt cascade by dephosphorylating the substrate of phosphoinositide 3-kinase, which phosphorylates Akt^21^. Reactive oxygen species, nitric oxide and endogenous electrophiles, such as Δ12-prostaglandin J_2_ and 4-hydroxynonenal, can activate the PTEN–Akt signaling pathway though modification of cysteine residues in PTEN, which has 10 cysteine residues (both in mouse, NP_032986, and in human, NP_000305)^22–25^. For example, hydrogen peroxide can oxidize PTEN to form a disulfide bond between Cys71 and Cys124, which are located close to one other^22, 26^. Numajiri *et al*. found that *S*-nitrosylation through Cys83 in PTEN regulated Akt signalling *in vivo* ^27^. Although the p*K*a value of cysteine is 8–9, the p*K*a value of the cysteine thiol proximal to basic amino acids, such as histidine, lysine and arginine, was decreased^2^. Of interest, Cys71, Cys83 and Cys124 are located near basic amino acids, including arginine and lysine, indicating that 1,4-NQ could potentially modify these cysteine residues. Consistent with this, we identified Cys71 and Cys83 as modification sites for 1,4-NQ (Fig. 2C), but did not detect modification of Cys124 under these conditions. Shearn *et al*. reported that 4-hydroxynonenal modified Cys71, but not Cys124, in PTEN^28^. This suggested that the organic electrophile had a preference for Cys71, rather than Cys124, in formation of the electrophile adducts detected by MS/MS.

Whereas 1,4-NQ exposure caused cytotoxicity and covalent modification of cellular proteins in primary mouse hepatocytes, the additional presence of Na_2_S_4_ blocked this protein modification, thereby protecting the cells from cytotoxicity (Fig. 1). In another study, we demonstrated that reactive persulfide and polysulfide species suppressed 1,4-NQ- and Cd-mediated activation, through electrophilic HSP90 modification, of HSP90–HSF1 signaling^18, 19^. In this study, we found that *S*-arylation of PTEN by 1,4-NQ was also suppressed by cotreatment with Na_2_S_4_ (Fig. 6). Because PTEN can negatively regulate the Akt cascade, we postulated that 1,4-NQ would activate the PTEN–Akt signaling pathway and that this activation could be modulated by polysulfides such as Na_2_S_4_. Consistent with these predictions, 1,4-NQ activated Akt and its downstream protein CREB in a concentration dependent manner, up to 10 µM, an effect suppressed by addition of Na_2_S_4_ (Fig. 3). At higher 1,4-NQ concentrations, however, Akt and CREB activation did not occur, indicating a bell-shaped dose response (Fig. 3). We obtained similar results in our previous study, in which MeHg, at lower concentrations, activated PTEN–Akt–CREB signaling and, at higher concentrations, disrupted the signaling because of nonspecific binding of MeHg to Akt and CREB^16^. Consistent with this, while nitric oxide, at lower concentrations, is an endogenous activator of Akt signaling through *S*-nitrosylation to PTEN, at higher concentrations nitric oxide can inactivate PTEN–Akt signaling, disrupting Akt function through *S*-nitrosylation^27^. Disruption of redox signaling through *S*-modification may protect cells by preventing overactivation of these pathways. Of interest, Na_2_S_4_ elevated the threshold of 1,4-NQ-mediated Akt and CREB phosphorylation in primary mouse hepatocytes, at least in part by suppression of 1,4-NQ-dependent *S*-modification of proteins, including PTEN, by the polysulfide (Figs 1C,D, 2A and 3). These observations suggested that the polysulfide Na_2_S_4_ modulated adaptive responses, such as activation of redox signaling caused by electrophilic modifications.

Incubation of 1,4-NQ with Na_2_S_4_ consumed 1,4-NQ^19^ and the reaction products formed were identical to 1,4-NQ–S–1,4-NQ-OH (Fig. 4). The polysulfide also reacted with MeHg to form MeHg-S-MeHg ((MeHg)_2_S), a detoxification metabolite of MeHg^29, 30^, suggesting that the 1,4-NQ-sulfur adducts we observed were also less cytotoxic. Thus, the authentic 1,4-NQ-sulfur adduct did not show any cytotoxicity or ability to covalently bind cellular proteins (Fig. 5A and B). Importantly, since even the sensor protein PTEN was not modified by the 1,4-NQ–sulfur adduct, PTEN–Akt–CREB signaling was not activated by this adduct (Fig. 5C and D). Inhibition of 1,4-NQ-mediated PTEN–Akt–CREB signaling by simultaneous exposure of primary mouse hepatocytes to this quinone and Na_2_S_4_ was at least partially caused by 1,4-NQ trapping by the polysulfide to form the 1,4-NQ–sulfur adduct in the culture medium (Fig. S4). We previously reported detection of a variety of sulfur adducts of environmental electrophiles, including an acrylamide-S-acrylamide adduct (Abiko Y *et al*., unpublished observations)^31^, 1,2-NQ–S–1,2-NQ adduct^32^, *tert*-butyl-1,4-benzoquinone–S–*tert*-butyl-1,4-benzoquinone adduct^32^, *N*-acetyl-*p*-benzoquinoneimine (NAPQI)–S–NAPQI adduct^33^, potentially unreactive to cellular nucleophiles like (MeHg)_2_S, and, now, the 1,4-NQ-sulfur adduct identified in this study. These results strongly indicated that highly reactive nucleophilic sulfur species, such as Na_2_S_4_, can scavenge environmental electrophiles to form their sulfur adducts that lack electrophilic characteristics.

In summary, previous studies showed that 1,2-NQ activated the Keap1–Nrf2 and PTP1B–EGFR signaling pathways through covalent binding to Cys151, Cys273, Cys288, Cys257 and Cys488 in Keap1^34^ and Cys121 in PTP1B^9^. Its isomer, 1,4-NQ, activated the HSP90–HSF1 pathway through covalent modification to Cys412 and Cys564 in HSP90^19^. The present study showed, in addition, that 1,4-NQ activated the PTEN–Akt signaling pathway through modification to Cys71 and Cys83 on PTEN. From these observations, it seems likely that environmental electrophiles at lower concentrations can activate redox signaling pathways by electrophilic modification of thiol groups in sensor proteins. This would result in adaptive responses useful for cell survival, cell proliferation, detoxification and excretion of electrophiles and quality control of cellular proteins. Reactive polysulfides can negatively regulate the quinone-mediated activation of redox signaling, such as the HSP90–HSF1 and PTEN–Akt pathways, by capturing environmental electrophiles to form inert sulfur adducts. Environmental electrophiles can react with not only Na_2_S_4_, the agent used in this study, but also endogenous per/polysulfides such as GSSH, GSSSG and CysSSH^19, 29, 35^. Endogenous H_2_S and persulfides/polysulfides are produced by enzymatic reaction of CSE, CBS, and 3-mercaptopyruvate sulfurtransferase^36–38^, which may play an important role on protection against electrophiles mediated toxicity. We showed that: 1) NAPQIH_2_–SSSCys and NAPQIH_2_–SSG adducts were detected in biological samples from mice given acetaminophen; 2) (MeHg)_2_S was produced from the reaction of MeHg with GSSH and/or GSSSG; and 3) 1,4-NQ reacted with Cys persulfide, and/or its polysulfide generated enzymatically by CSE, to yield 1,4-NQ–SCys, 1,4-NQ–SH, and 1,4-NQ–S–1,4-NQ adducts^19^. Taken together, these findings suggested that reactive per/polysulfide species have the potential to modulate the adaptive responses caused by environmental electrophile exposures. Thus, supplementation or other simultaneous intake of per/polysulfide species might decrease the health risks of environmental electrophile exposures.

## Methods

### Materials

Dimethyl sulfoxide (DMSO), 1,4-NQ (98% purity determined by gas chromatography) and anti-GAPDH antibody were from Wako Pure Chemical Industries (Osaka, Japan), Tokyo Chemical Industries (Tokyo, Japan) and Santa Cruz Biotechnology (Santa Cruz, CA, USA), respectively. Biotin-PEAC_5_-maleimide (BPM) and Na_2_S_4_ were from Dojindo Laboratories (Kumamoto, Japan). Dynabeads M-280-sheep anti-rabbit immunoglobulin G (IgG) was from Invitrogen (Carlsbad, CA, USA). Anti-Akt, anti-CREB, anti-phosphorylated Akt (Ser473), anti-phosphorylated CREB (Ser133), horseradish peroxidase (HRP)-conjugated anti-biotin antibodies, anti-rabbit antibodies and anti-mouse IgG secondary antibodies were from Cell Signaling Technology (Beverly, MA, USA). *Escherichia coli* BL21 cells and trypsin were from Promega Co. (Madison, WI, USA). Glutathione 4B Sepharose was from GE Healthcare (Chicago, IL, USA). All other reagents were of the highest purity available.

### Isolation and culture of primary mouse hepatocytes

All animal protocols were approved by the University of Tsukuba Animal Care and Use Committee and were performed strict adherence to the committee’s guidelines for alleviation of suffering. Primary mouse hepatocytes were isolated from 6–11-wk-old C57BL/6 J female mice as described previously^39^. Briefly, the hepatocytes (8 × 10^4^ cells/cm^2^) were seeded in William’s medium E containing 10% fetal bovine serum, 2 mM glutaMAX-I (Thermo Fisher Scientific, Waltham, MA, USA) and antibiotics (100 units/mL penicillin and 100 µg/mL streptomycin) on culture plates coated with fetal bovine type I collagen (Corning Inc., Corning, NY, USA) and were maintained at 37 °C in a humidified atmosphere containing 95% air and 5% CO_2_. The cells were cultured for 2 d after isolation and then starved overnight by incubation in serum-free medium before exposure to 1,4-NQ.

### Lysate preparation

After exposure to 1,4-NQ, with or without Na_2_S_4_, primary mouse hepatocytes were washed twice with ice-cold phosphate-buffered saline. A cell lysate was then prepared by sonicating the cells in radioimmunoprecipitation assay (RIPA) buffer [25 mM Tris-HCl (pH 7.5), 150 mM sodium chloride, 1% NP40 and 0.5% sodium deoxycholic acid] containing 1% protease inhibitor cocktail (Sigma-Aldrich, St. Louis, MO, USA). The cells lysed in RIPA buffer were centrifuged for 10 min at 14,000 *g*. Protein concentrations were determined using the bicinchoninic acid assay (Thermo Fisher Scientific).

### Western blot analysis

Samples, adjusted for equal protein contents, were each mixed with a half volume of SDS-PAGE loading buffer [62.5 mM Tris-HCl (pH 6.8), 8% glycerol (v/v), 2% SDS (w/v) and 0.005% bromophenol blue (w/v)] containing either 15 mM 2-mercaptoethanol or 50 mM tris(2-carboxyethyl)phosphine. Each mixture was then heated to 95 °C for 5 min and applied to a SDS-polyacrylamide gel. The proteins were separated by SDS-PAGE and electro-transferred onto polyvinylidene difluoride membranes (Bio-Rad Laboratories, Hercules, CA, USA) at 2 mA/cm^2^ for 1 h. The membranes were blocked in 5% skim milk at 25 °C for 1 h, incubated with primary antibodies at 4 °C overnight and then incubated with secondary antibodies coupled to HRP at room temperature for 2 h. Stained protein bands were detected using an enhanced chemiluminescence system (Nacalai Tesque, Kyoto, Japan) using a LAS 3000 imager (Fujifilm, Tokyo, Japan).

### Preparation of recombinant PTEN

The entire coding sequence of human wildtype PTEN was amplified from a human cDNA library by the polymerase chain reaction (PCR). The cDNA encoding PTEN was subcloned into the pGEX-6P-1 vector. The recombinant human PTEN was expressed as an N-terminal glutathione *S*-transferase (GST)-tagged fusion protein in BL21 (DE3) cells transformed with the pGEX-PTEN vector. Protein production was induced by 0.5 mM isopropyl β-D-thiogalactopyranoside (Nacalai Tesque) at 25 °C for 12 h. GST-PTEN was affinity purified on Glutathione 4B Sepharose, eluted with 10 mM reduced glutathione in 50 mM Tris-HCl (pH 7.5), 150 mM sodium chloride and 1 mM dithiothreitol (DTT). Thiol groups oxidized during purification were reduced by incubation with 20 mM DTT for 1 h. Free glutathione and DTT were removed by buffer exchange to 50 mM potassium phosphate buffer (pH 7.0) using an ultrafiltrator (Piece Concentrators 9 K; Thermo Fisher Scientific). Samples were stored in at −80 °C before use.

### Detection of cellular PTEN modified by 1,4-NQ

Primary mouse hepatocytes were exposed to 1,4-NQ (10 µM) for 30 min, with or without Na_2_S_4_, then lysates were prepared using RIPA buffer, as described above under lysate preparation. Anti-rabbit IgG conjugated magnetic beads (100 µL, Dynabeads M-280-sheep anti-rabbit IgG) were washed three times with Tris-buffered saline and Tween 20, then incubated with anti-PTEN antibodies (5 µL, Cell Signaling Technology, #9552) at 4 °C for 3 h. The unbound antibodies were removed and the beads resuspended in 500 µL cell lysate (1 µg/µL). The mixture was then incubated, with rotation, at 4 °C overnight. The beads were then washed four times with RIPA buffer and protein complexes eluted by adding 40 µL RIPA buffer and a half volume of SDS-PAGE loading buffer containing 50 mM tris(2-carboxyethyl)phosphine. The eluted proteins were incubated at 95 °C for 5 min, then analyzed by western blotting.

### Recombinant PTEN modification and LC-MS^E^ analysis

Recombinant GST-PTEN (1 μg) was incubated with 1,4-NQ at 25 °C for 1 h. The reaction mixture was then analyzed by western blotting with an anti-1,4-NQ antibody. For LC-MS analysis, 1.7 μg protein was incubated with 1,4-NQ (10 μM) at 25 °C for 30 min in a total volume of 10 μL 50 mM Tris-HCl (pH 7.5). Samples of native and 1,4-NQ-modified GST-PTEN were incubated with 2 mM tris(2-carboxyethyl)phosphine at 25 °C for 10 min in a total volume of 20 μL 50 mM ammonium bicarbonate solution. Each mixture was then alkylated by adding 2.5 μL 30 mM 2-iodoacetamide in 50 mM ammonium bicarbonate solution and incubating the mixture at 25 °C for 20 min in the dark. The GST-PTEN was digested by adding 2.5 μL MS-grade modified trypsin (100 ng) and incubating the mixture at 37 °C overnight. Nano UPLC-tandem MS (MS^E^) analysis was performed using a nanoAcquity UPLC system (Waters, Milford, MA, USA), equipped with a BEH130 nanoAcquity C_18_ column (100 mm long, 75 μm i.d., 1.7 μm particle size; Waters), maintained at 35 °C. The analysis was performed in direct injection mode. Mobile phases A (0.1% formic acid) and B (acetonitrile with 0.1% formic acid) were mixed using a gradient system, at a flow rate of 0.3 μL/min. The mobile phase program started at 3% B for 1 min, then linearly increased over 74 min to 40% B, which was maintained for 4 min, then linearly increased over 1 min to 95% B, which was maintained for 5 min, then linearly decreased over 1 min to 3% B. The total run time (including conditioning the column at the initial conditions) was 100 min. The eluted peptides were transferred to the nano-electrospray source of a quadrupole time-of-flight mass spectrometer (a Synapt High Definition Mass Spectrometry system; Waters) through a Teflon capillary union and a precut PicoTip (Waters). The initial Synapt mass spectrometer parameters were capillary voltage of 2.8 kV, sampling cone voltage of 35 V and source temperature of 100 °C. A low (6 eV) or elevated (stepped from 15 to 30 eV) collision energy was used to generate either intact peptide precursor ions (low energy) or peptide product ions (elevated energy). The detector was operated in positive ion mode. The mass spectrometer performed survey scans from *m*/*z* 50 to 1990. All analyses were performed using an independent reference, glu-1-fibrinopeptide B (*m/z* 785.8426), which was infused through the NanoLockSpray ion source and sampled every 10 s and used as an external mass calibrant. Data were collected using MassLynx version 4.1 software (Waters). Biopharmlynx version 1.2 software (Waters) was used to perform baseline subtraction, smoothing, de-isotoping, *de novo* peptide sequence identification and database searches.

### Synthesis of the reaction products of 1,4-NQ and Na_2_S_4_

1,4-NQ (31.6 mg) was dissolved in DMSO (4 mL), then incubated with Na_2_S_4_ (69.7 mg) dissolved in water (36 mL) for 10 min at room temperature. The resulting solution was separated by preparative column chromatography using an Ultra Pack ODS-SM-50C (30 × 37 mm i.d., 50 µm, Yamazen, Osaka, Japan), eluted with 20% acetonitrile for 40 min, followed by 80% acetonitrile for 60 min at a flow rate of 10 mL/min. Each fraction was characterized by UV absorbance at 250 nm and UPLC-MS analysis. The fractions containing the product of *m/z* 361 in negative ion mode were collected and applied to the same column again and eluted with 15% acetonitrile for 50 min at a flow rate of 10 mL/min. The fractions containing the purified product of *m/z* 361 in negative ion mode were collected and then evaporated to remove acetonitrile in the solution. The resulting solution was lyophilized to yield a dark-orange powder. ^1^H NMR and ^13^C NMR analysis were performed on the isolated compound on a Bruker 600 MHz NMR spectrometer, using DMSO-d6 as the solvent. ^1^H NMR (600 MHz, DMSO-d6): δ 8.05 (d, J = 3.7 Hz, 1 H), 7.97 (d, J = 3.6 Hz, 1 H), 7.93 (d, J = 3.7 Hz, 1 H), 7.91 (d, J = 5.8 Hz, 1 H), 7.86 (t, J = 7.4 Hz, 1 H), 7.83 (t, J = 7.4 Hz, 1 H), 7.74 (t, J = 7.5 Hz, 1 H) and 7.63 (t, J = 7.5 Hz, 1 H), 6.07 (s, 1 H). ^13^C NMR (600 MHz, DMSO-d6): δ 183.5, 182.9, 180.8, 177.3, 171.9, 154.7, 135.6, 134.4, 133.7, 133.2, 131.9, 131.7, 131.4, 130.8, 126.5, 126.0, 125.79, 125.77, 125.6 and 99.3.

### Characterization of the 1,4-NQ sulfur adduct by UPLC-MS

UPLC-MS^E^ analysis was performed using an Acquity UPLC system (Waters) equipped with an Acquity UPLC BEH C_18_ column (2.1 mm × 50 mm i.d., 1.7 µm) maintained at 35 °C. Mobile phase A (0.1% formic acid) and B (100% acetonitrile with 0.1% formic acid) were linearly mixed, at a flow rate of 0.3 ml/min, using the following gradient system: 20% B for 2 min with a linear increase over 7 min to 90% B. The total running time, including the initial conditioning of the column, was 15 min and the injection volume was 10 µL. The eluted compounds were then transferred to the photodiode array (PDA) detector and the electrospray source of the Synapt HDMS system. Electron spray ionization (ESI) was used, with a capillary voltage of 2.5 kV, sampling cone voltage of 30 V, transfer cone voltages of 4 V. Low (6 eV) or elevated (steps from 20–30 eV) collision energies were used to generate either the intact precursor ions (low energy) or the product ions (elevated energy). The source temperature was 90 °C, and the detector was operated in negative ion mode. Data were collected from *m/z* 50 to 1000. These data were acquired using an independent reference spray via LockSpray interference with leucine enkephalin [M‒H]^‒^ ion as the lock mass (*m/z* 554.26) to ensure accuracy and reproducibility. Data were analyzed with MassLynx version 4.1 software.

### Fourier transform ion cyclotron resonance mass spectrometry (FT-ICR-MS)

MS spectra were obtained using a Bruker Solarix XR 7.0 T (Bruker Daltonics, Bremen Germany). The sample solution was introduced through an infusion pump at a flow rate of 120 µL/h. Measurement conditions were: dry N_2_ gas temperature, 200 °C; ESI, negative mode; capillary voltage, 4500 V. Data were collected from *m/z* 100 to 1000.

### BPM assay

BPM-labeling assay was performed as described previously^40, 41^. Briefly, primary mouse hepatocytes were exposed to 1,4-NQ or 1,4-NQ–S–1,4-NQ-OH, at molar equivalent concentrations of 1,4-NQ (40 µM), for 1 h, then lysates were prepared using RIPA buffer, as described under lysate preparation. The cell lysates were incubated with BPM (100 µM) at 37 °C for 30 min. Recombinant GST-PTEN (1 μg) was incubated with quinones, at molar equivalent concentrations of 1,4-NQ (10 µM), at 25 °C for 1 h and then further incubated with BPM (15 μM) at 37 °C for 30 min. The samples were mixed with a half volume of SDS-PAGE loading buffer containing 50 mM tris(2-carboxyethyl)phosphine, incubated at 95 °C for 5 min and analyzed by western blotting using an HRP-conjugated anti-biotin antibody. Total protein content was also assessed by SDS-PAGE with Coomassie Brilliant Blue staining.

### Cellular viability

Cellular toxicities of 1,4-NQ or related compounds were estimated using the 3-(4,5-dimethythiazol-2-yl)-2,5-triphenyl tetrazolium bromide (MTT) assay, as described previously^42^. Briefly, primary mouse hepatocytes on 96-well plates were exposed to chemicals for 24 h, then incubated with 5 mg/mL MTT for 3 h at 37 °C. The medium was removed and 100 μL DMSO was added to dissolve the formazan. The absorbance of samples at 540 nm was determined using an iMark microplate reader (Bio-Rad Laboratories).

### Statistical analysis

All data are expressed as means ± standard error for at least three independent experiments. Statistical significance was assessed by one-way ANOVA followed by Tukey’s post-hoc test using KaleidaGraph (Synergy Software, Reading, PA, USA), *P* < 0.05 or *P* < 0.01 were considered significant.

## Electronic supplementary material

Supplementary Information
