# Supplementary material for: Polysulfide Na2S4 regulates the activation of PTEN/Akt/CREB signaling and cytotoxicity mediated by 1,4-naphthoquinone through formation of sulfur adducts
Source: Sci Rep. 2017 Jul 6;7:4814. doi: 10.1038/s41598-017-04590-z (PMC5500523; doi:10.1038/s41598-017-04590-z)
Supplement: Supplementary file 1 — Supplementary Information [file 41598_2017_4590_MOESM1_ESM.pdf]

Supplemental materials

Polysulfide Na_2S_4 regulates the activation of PTEN/Akt/CREB signaling and cytotoxicity mediated by 1,4-naphthoquinone, through formation of sulfur adducts

Yumi Abiko[a, #], Yasuhiro Shinkai[a, #], Takamitsu Unoki[a], Reiko Hirose[a], Takashi Uehara[b], Yoshito Kumagai[a,*]

[a] Faculty of Medicine, University of Tsukuba, Tsukuba, Ibaraki 305-8575, Japan
[b] Department of Medicinal Pharmacology, Graduate School of Medicine, Dentistry, and Pharmaceutical Sciences, Okayama University, Okayama 700-8530, Japan
# Y.A and Y.S. contributed equally in this study

[*] Corresponding author. Yoshito Kumagai, Faculty of Medicine, University of Tsukuba, 1-1-1 Tennodai, Tsukuba, Ibaraki 305-8575, Japan
Tel: +81-29-853-3297; Fax: +81-29-853-3259; E-mail yk-em-tu@md.tsukuba.ac.jp

Table of contents

Supplementary Figure S1. 2
Supplementary Figure S2. 3
Supplementary Figure S3. 4
Supplementary Figure S4. 5

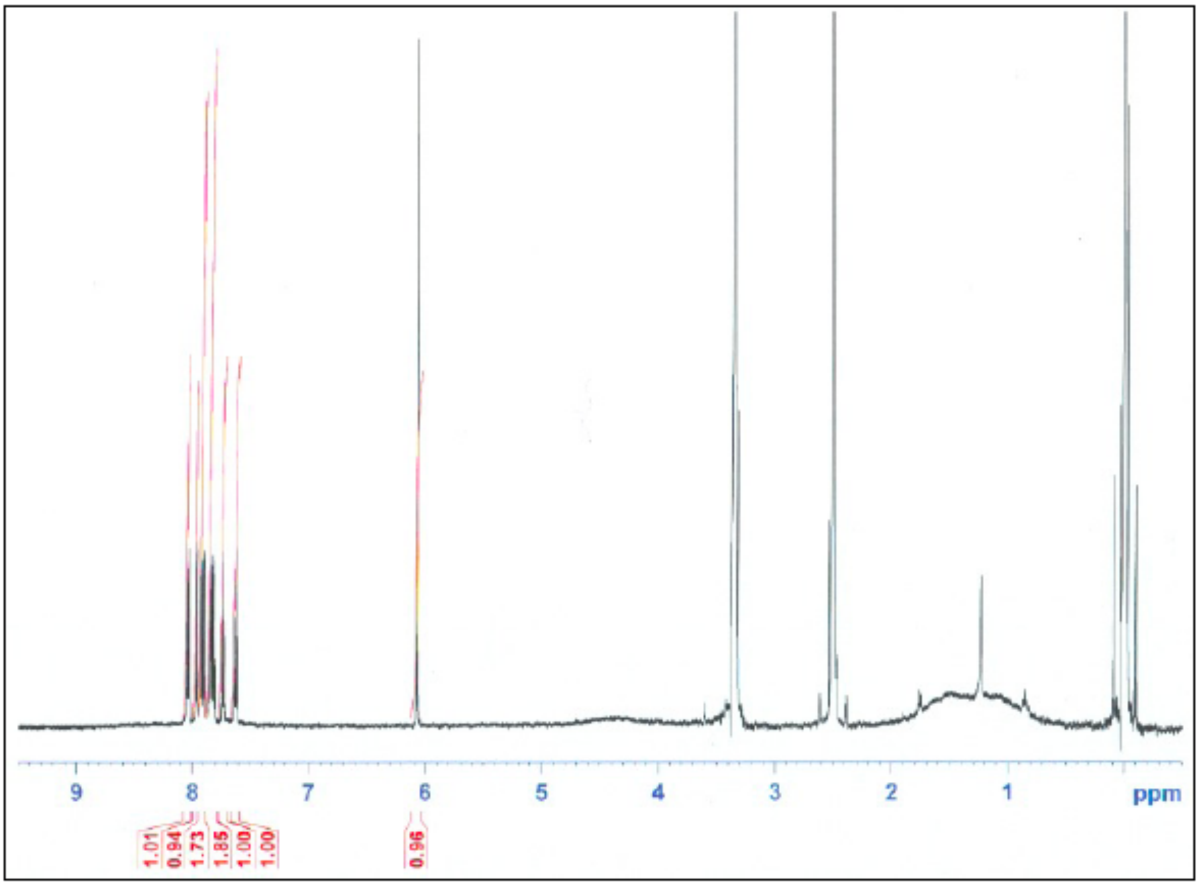

Figure S1. ^{1}H NMR spectrum of a sulfur adduct of 1,4-NQ.

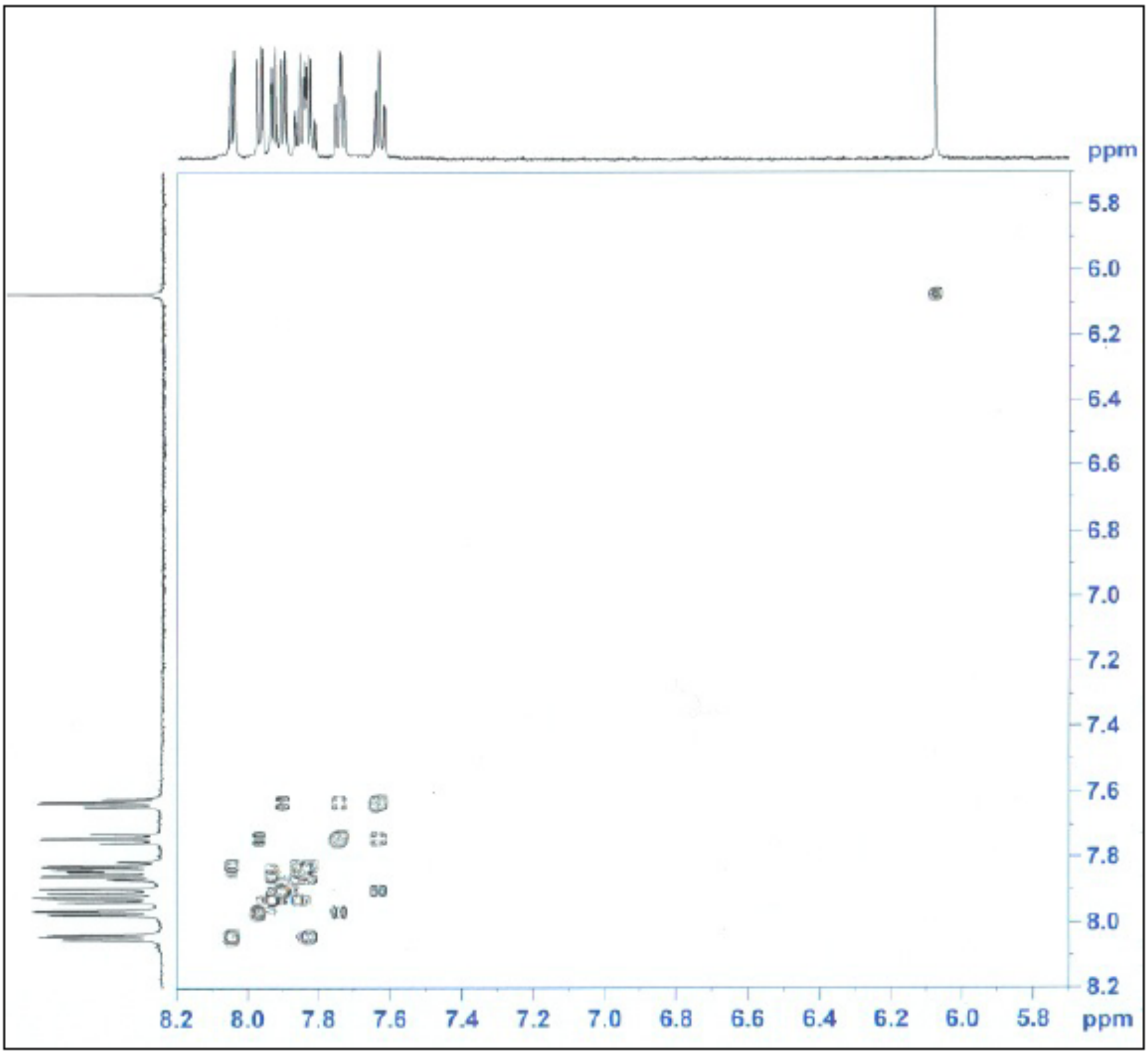

Figure S2. ^{1}H-^{1}H COSY NMR spectrum of a sulfur adduct of 1,4-NQ.

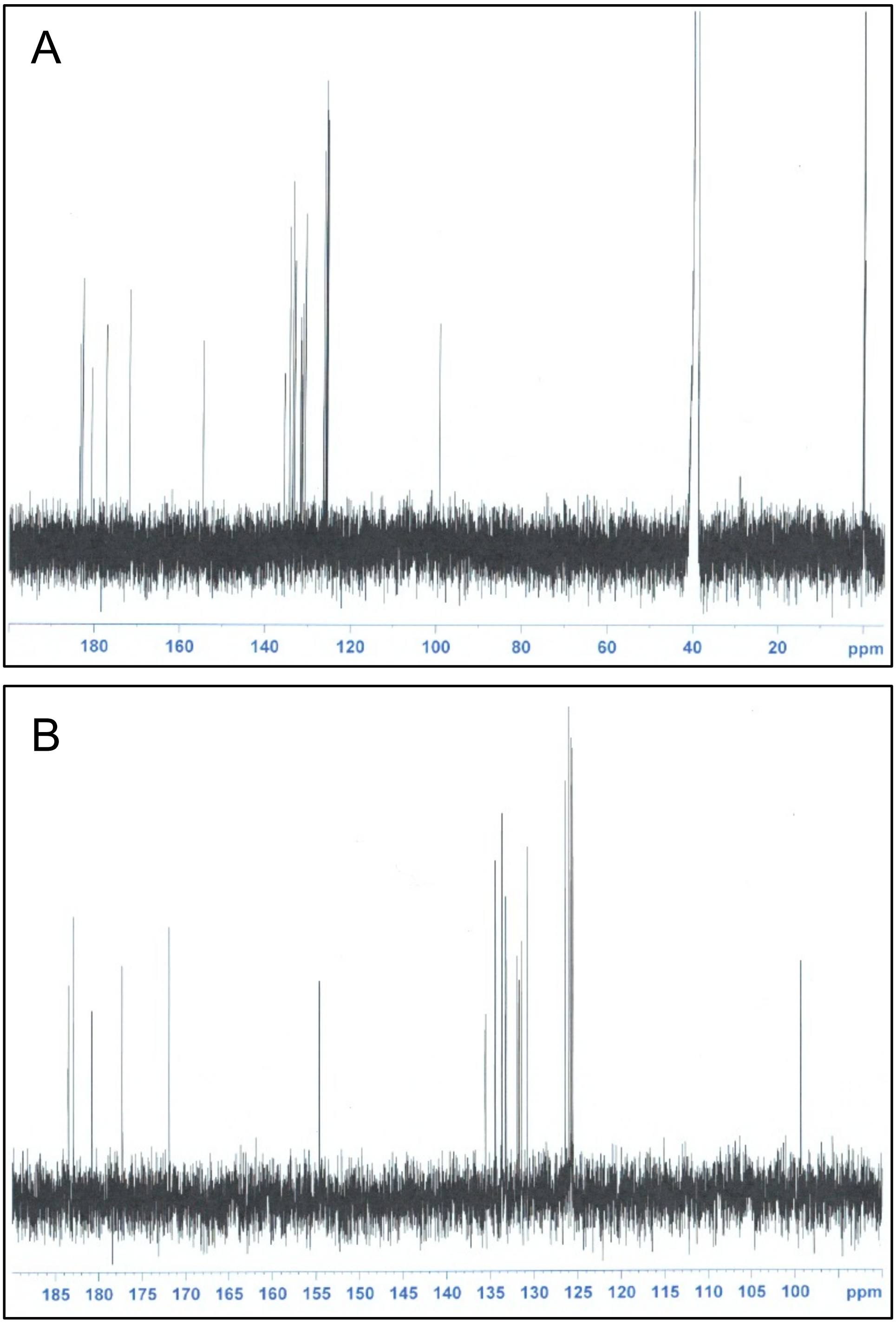

Figure S3. ^{13}C NMR spectrum of a sulfur adduct of 1,4-NQ. ^{13}C NMR spectrum (A) and a magnification of spectrum A (B) are shown.

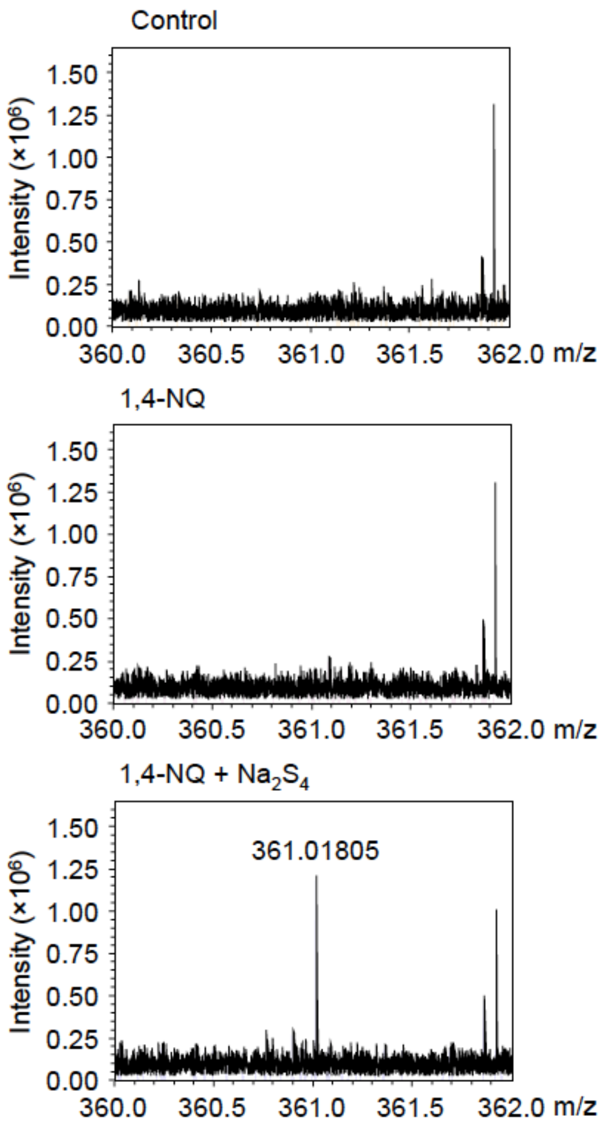

Figure S4. Detection of 1,4-NQ sulfur adducts in the culture medium. Mouse primary hepatocytes were exposed to 1,4-NQ (40 μM) with or without 100 μM Na_2S_4 for 30 min, then 1,4-NQ sulfur adducts in the culture medium were detected by FT-ICR-MS.
